# Cost-benefit analysis of introducing next-generation sequencing (metagenomic) pathogen testing in the setting of pyrexia of unknown origin

**DOI:** 10.1371/journal.pone.0194648

**Published:** 2018-04-17

**Authors:** Jia Hui Chai, Chun Kiat Lee, Hong Kai Lee, Nicholas Wong, Kahwee Teo, Chuen Seng Tan, Praveen Thokala, Julian Wei-Tze Tang, Paul Anantharajah Tambyah, Vernon Min Sen Oh, Tze Ping Loh, Joanne Yoong

**Affiliations:** 1 Saw Swee Hock School of Public Health, National University of Singapore, Singapore, Singapore; 2 Department of Laboratory Medicine, National University Hospital, Singapore, Singapore; 3 Department of Clinical Microbiology, University Hospital of Leicester NHS Trust, Leicester, United Kingdom; 4 Department of Paediatrics, University Hospital of Leicester NHS Trust, Leicester, United Kingdom; 5 Health Economics and Decision Science, School of Health and Related Research, The University of Sheffield, Sheffield, United Kingdom; 6 Department of Infection, Immunity, Inflammation, University of Leicester, Leicester, United Kingdom; 7 Department of Medicine, National University of Singapore, Singapore, Singapore; The University of Hong Kong, HONG KONG

## Abstract

Pyrexia of unknown origin (PUO) is defined as a temperature of >38.3°C that lasts for >3 weeks, where no cause can be found despite appropriate investigation. Existing protocols for the work-up of PUO can be extensive and costly, motivating the application of recent advances in molecular diagnostics to pathogen testing. There have been many reports describing various analytical methods and performance of metagenomic pathogen testing in clinical samples but the economics of it has been less well studied. This study pragmatically evaluates the feasibility of introducing metagenomic testing in this setting by assessing the relative cost of clinically-relevant strategies employing this investigative tool under various cost and performance scenarios using Singapore as a demonstration case, and assessing the price and performance benchmarks, which would need to be achieved for metagenomic testing to be potentially considered financially viable relative to the current diagnostic standard. This study has some important limitations: we examined only impact of introducing the metagenomic test to the overall diagnostic cost and excluded costs associated with hospitalization and makes assumptions about the performance of the routine diagnostic tests, limiting the cost of metagenomic test, and the lack of further work-up after positive pathogen detection by the metagenomic test. However, these assumptions were necessary to keep the model within reasonable limits. In spite of these, the simplified presentation lends itself to the illustration of the key insights of our paper. In general, we find the use of metagenomic testing as second-line investigation is effectively dominated, and that use of metagenomic testing at first-line would typically require higher rates of detection or lower cost than currently available in order to be justifiable purely as a cost-saving measure. We conclude that current conditions do not warrant a widespread rush to deploy metagenomic testing to resolve any and all uncertainty, but rather as a front-line technology that should be used in specific contexts, as a supplement to rather than a replacement for careful clinical judgement.

## Introduction

Pyrexia of unknown origin (PUO) is defined as a temperature of more than 38.3°C that lasts for more than three weeks, where no cause can be found despite appropriate investigation [1]. Existing protocols for the work-up of PUO can be extensive and costly, motivating the application of recent advances in molecular diagnostics to pathogen testing. Metagenomic pathogen testing or unbiased next-generation sequencing (NGS) sequences all genetic material in a clinical sample, including those of the pathogens [2], enabling the detection of pathogens that may be otherwise missed by current conventional approaches such as targeted polymerase chain reaction (PCR) or sequencing methods.

The performance of NGS has been widely documented and its clinical potential demonstrated [2–20]. In a recent illustrative case, a boy with severe combined immunodeficiency was admitted recurrently for PUO and neurological symptoms, with no actionable cause identified after weeks of extensive investigation [3]. Finally, metagenomic pathogen testing of the patient’s cerebrospinal fluid revealed genetic sequences matching *Leptospiraceae* family were detected, leading to diagnosis of neuroleptospirosis, prompt administration of appropriate antimicrobial therapy, and full recovery.

The economics of NGS has been less well studied, although this is a critical first step towards widespread adoption into clinical practice. From a cost perspective, NGS may help to avert second- or third-line investigations and therefore, in principle, for applications with a sufficiently high detection rate, cost savings may be realized that can offset the cost of the test. However, in practice, the case for deploying this relatively expensive diagnostic modality is not always achievable and requires a systematic consideration of the likely costs and benefits.

This study pragmatically evaluates the feasibility of introducing NGS in this setting by assessing the relative cost of clinically-relevant strategies employing this investigative tool under various cost and performance scenarios using Singapore as a demonstration case, and assessing the price and performance benchmarks which would need to be achieved for NGS to be potentially considered financially viable relative to the current diagnostic standard. We discuss the implications and limitations of our findings for clinicians and health system decision makers considering when and if to adopt NGS for adult PUO, and more broadly for future translational research.

## Methods

**Fig 1:**
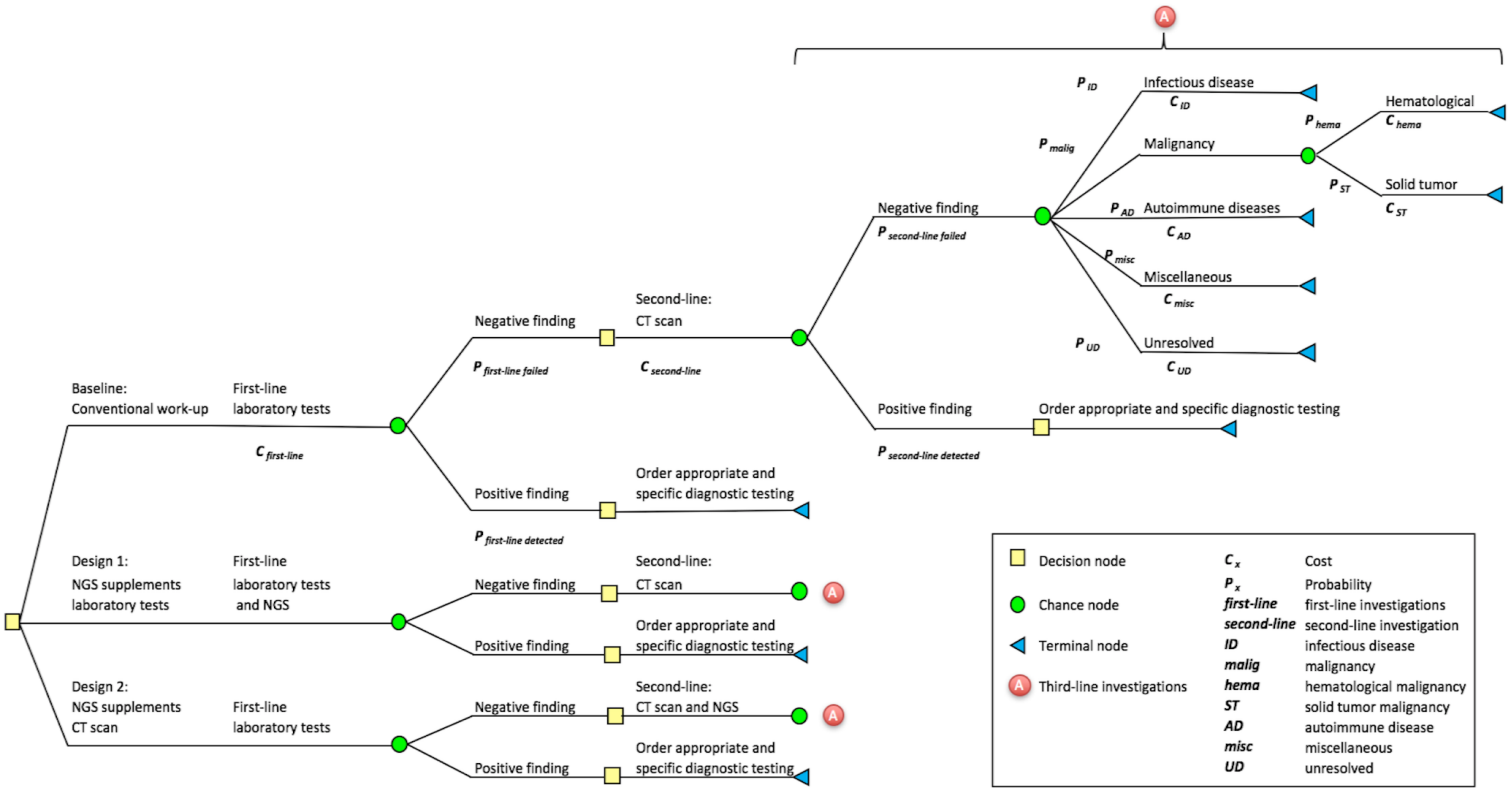
Decision tree representing the conventional diagnostic workflow for PUO in adults based on a clinical guideline [1], and the subsequent introduction of metagenomic pathogen testing as first- or second-line investigations.

To systematically describe the costs and benefits associated with introducing NGS relative to existing standard of care, we built and parameterised a decision tree model that captures the stylized diagnostic workflow based on published guidelines for the investigation of adult PUO [1] as shown in Fig 1 as well as a counterfactual scenario using NGS based on expert opinion. As data availability is limited in this setting, several assumptions based on published literature or expert opinion, were used to further simplify model construction and populate the decision tree. We then computed the diagnostic workflow: Standard of care vs. first/ second-line NGS.

Briefly, the existing diagnostic workflow starts with detailed clinical history and physical examination of the patient. Following this, a battery of first-line laboratory and radiologic investigations are performed (Table 1). If these first-line tests are negative, a second-line investigation (computerized tomography scan) is performed. The combined results of the first- and second-line investigations then guide subsequent third-line (disease-specific) investigations (Fig 1). Of note, positron emission tomography scan was assumed to be performed routinely in the investigation of PUO, according to a more recent recommendation [21]. We assumed all investigations along each diagnostic pathway are performed until a first- or second-line investigation was positive i.e. a diagnosis is reached.

**Table 1:** Investigations recommended for work-up of PUO in the clinical guideline [1] with modifications. The total cost for each line of investigations are shown in Singapore dollars (SGD).

Investigations	Total cost
**First-line (non-specific) investigations**	SGD 826.30
Complete blood count with differential	
Electrolyte panel	
Liver function tests	
Gamma-glutamyl transferase	
Blood cultures x 3	
Urine dipstick	
Urine microscopy	
Urine cultures	
Erythrocyte sedimentation rate	
Quantiferon test for tuberculosis	
Chest radiography	
**Second-line investigation**	SGD 850.00
Computed tomography scan of abdomen and pelvis	
**Third-line (disease-specific) investigations**	
*Infectious diseases*	SGD 433.00
Urine culture	
Sputum culture and gram stain	
Sputum culture for tuberculosis	
VDRL	
Epstein-Barr virus IgG	
Epstein-Barr virus IgM	
Cytomegalovirus IgG	
Cytomegalovirus IgM	
Human immunodeficiency virus serology	
Antistreptolysin-O antibodies titer	
*Malignancies (hematological)*	SGD 56.10
Peripheral blood smear	
Serum protein electrophoresis	
*Malignancies (solid tumor)*	SGD 2840.00
Bone scan	
Mammography	
Chest CT with contrast	
Positron emission tomography scan	
*Autoimmune disease*	SGD 40.00
Rheumatoid factor	
Antinuclear antibodies	
*Miscellaneous*	SGD 158.20
Thyroid studies	
Angiotensin-converting enzyme levels (ACE)	
*Unresolved diagnosis*	SGD 3527.30
Sum of costs for all third-line, disease-specific investigations above	

As an alternative, we model two likely strategies for the introduction of NGS as part of these guidelines, i.e. as adjunct first- or second-line investigations (Fig 1). In each case, the metagenomic tests were assumed to be performed at the same time as the first- or second-line tests, with the collective results made available before the next line of investigations are ordered. Any positive detection by the metagenomic test was assumed to be definitive under these guidelines and no further investigations would be conducted.

### Probabilities of diagnosis and unit costs

In the baseline scenario without NGS, the probability of any first-line investigations finding a specific diagnosis was assumed to be 10%, based on local clinical expert opinion and a prospective multi-center study of first-line laboratory and radiology tests in the setting of PUO [22]. The probability of the computed tomography scan of abdomen and pelvis obtaining a specific diagnosis conditional on a negative first-line investigation was assumed to be 20%, based again on local clinical expert opinion and a range of 5–20% previously reported in the literature(Fig 1) [23]. Conditional probabilities of disease-specific diagnosis for each investigation pathway in PUO were not available due to data scarcity; proxy estimates were drawn from the averaged probabilities of 26 recently published studies summarized in a recent systematic review (Table 2) and reviewed by local clinicians for face validity [24]. The proportions of hematologic and solid tumor malignancy were assumed to be 29% and 71%, respectively, as reported previously by Sørensen et al [25].

**Table 2:** Probability of disease-specific diagnostic outcome (without next-generation sequencing).

Disease-specific diagnosis	%	Reference
**Baseline scenario**		
Infectious disease	36	Hayakawa et al 2012 [24]
Malignancies	13	Hayakawa et al 2012 [24]
Autoimmune disease	21	Hayakawa et al 2012 [24]
Miscellaneous	6	Hayakawa et al 2012 [24]
Unresolved diagnosis	24	Hayakawa et al 2012 [24]
**Low infectious disease scenario**		
Infectious disease	14	Vanderschueren et al 2003 [26]
Malignancies	11	Vanderschueren et al 2003 [26]
Autoimmune disease	21	Vanderschueren et al 2003 [26]
Miscellaneous	10	Vanderschueren et al 2003 [26]
Unresolved diagnosis	44	Vanderschueren et al 2003 [26]

Unit costs for current diagnostictests and procedures (Table 1) were obtained from the National University Hospital, Singapore and the Ministry of Health, Singapore, in 2016 Singapore dollars (SGD 1 = USD 0.71, SGD 1 = GBP 0.57, SGD 1 = Euro 0.67).

NGS is characterised by three empirically unknown parameters in our model: the cost of the test, the first-line detection rate and the second-line detection rate. As no test cost or detection rate data are publicly available, in the baseline scenario with NGS, we make the (arbitrary) initial assumption that detection rates 10% for first- and 20% for second-line investigations. We vary the detection rates for first- and second-line investigations in our sensitivity analysis over a wide range (0–100%). We also assume a wide range of hypothetical costs (SGD 100–1000) and assumed that all costs are variable. All the parameters for baseline scenario and variations are shown in Table 3.

**Table 3:** List of parameters in break-even analysis.

Parameters	Baseline	Low	High
Positive findings of first-line investigation	10%	5%	20%
Positive findings of second-line investigation	20%	10%	40%
Cost of next-generation sequencing	$1000	$100	$2000
Expected total cost of current standard of care	$2516	$2060	$3427

### Cost-benefit analysis

Our decision model compared the cost of alternative diagnostic strategies for a hypothetical patient in Singapore and determined the incremental cost or savings from introducing NGS in a two-step process. Firstly, for a given combination of the three test parameters (cost, first- and second-line detection rate), the expected value of the overall diagnostic cost for an episode of PUO was calculated for each strategy (no testing, first-line or second-line), by rolling back i.e. multiplying the probabilities of detection by the unit costs of the investigation at each relevant branch of the decision tree. No further discounting or price adjustment was applied as it was assumed all investigations take place within a calendar year. The optimal NGS-based strategy (first- or second-line) was determined based on cost-minimization. Subsequently, the expected value of introducing NGS was then computed by subtracting the expected cost of the optimal NGS-based strategy from the expected cost under current practice.

A break-even cost analysis was then conducted. The break-even price for NGS conditional on specific first- and second-line detection rate was defined as the highest price that results in the optimal NGS-based strategy being equal to the cost under current practice, and similarly the break-even first- (second-) line detection rate conditional on a specific price and second- (first-) line detection rate was defined as the lowest rate that results in the optimal NGS-based strategy being equal to the cost under current practice.

In general, for any given test cost, the overall diagnostic costs reduce with increasing detection rate of first- or second-line investigations since fewer patients require follow-up investigations, and for any given level of test performance, the incremental cost of introducing NGS increases with test price. At any price, the optimal NGS-based strategy depends on the relative performance of first- and second-line testing. The break-even cost of introducing NGS testing therefore generally increases with overall test performance, but this increase may in theory be nonlinear depending on whether there is a switch in optimal strategy from first- to second-line. We computed all combinations of price and detection rates that would enable the NGS to break even in the baseline scenario.

### Sensitivity analysis

In addition to the varying test parameters as described above, the impact of introducing the metagenomic test in a PUO setting with low prevalence of infectious disease was modelled as an alternative scenario, based on data from Vanderschueren et al [26] (Table 2). To further account for parameter uncertainty in costs for infectious disease, malignancies (solid tumour) and unresolved diagnosis, we further varied the total costs of these investigations by reducing 50% and increasing 100%.

All analyses were performed using Microsoft Office Excel 2011. The break-even analysis was conducted using the Data Table function.

## Results

**Fig 2:**
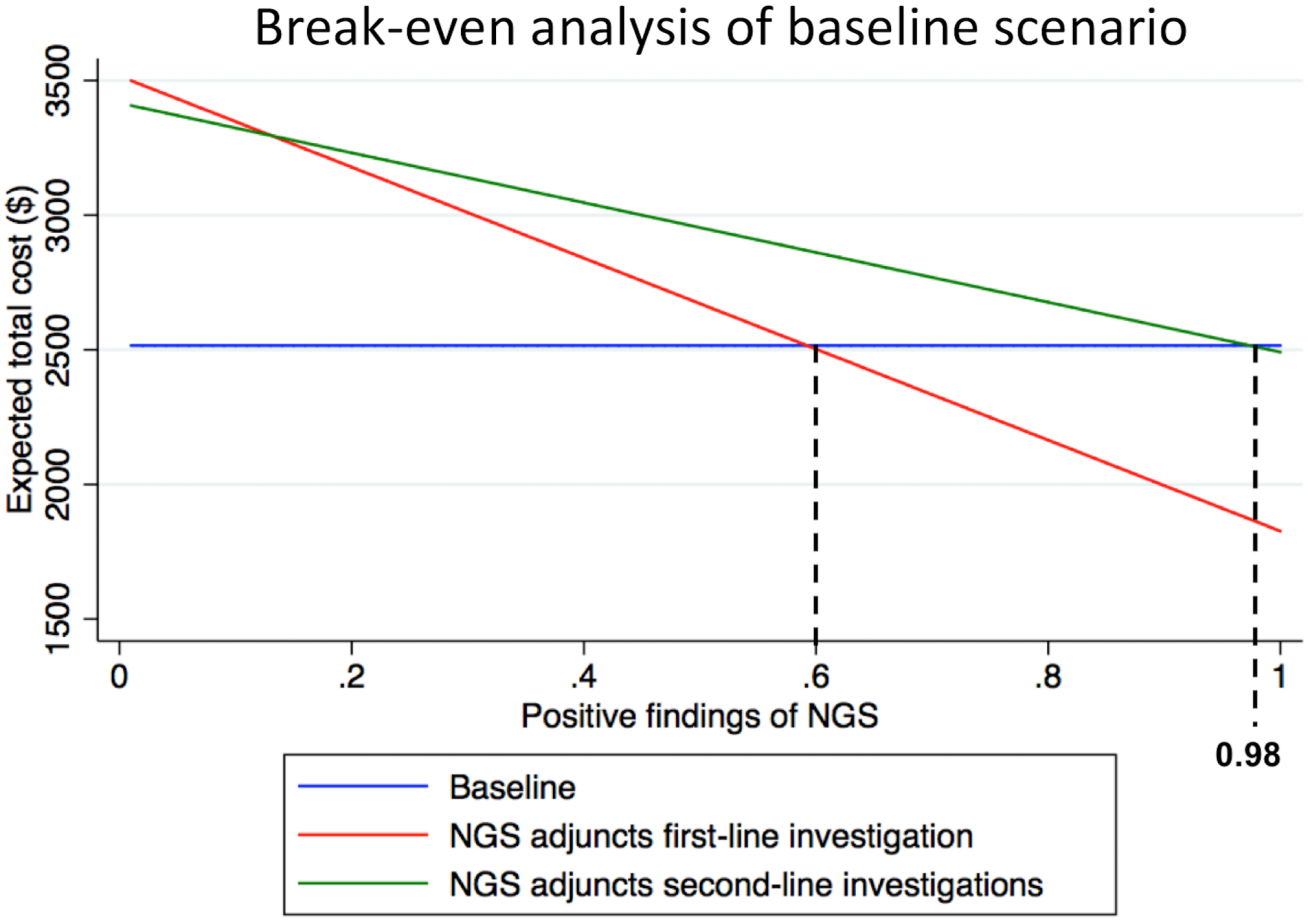
Expected cost of next-generation sequencing (NGS) strategies vs. current standard of care (baseline scenario).

For the baseline scenario, the cost implications for the different strategies for investigating patients with PUO and the computation of the break-even cost by varying first- and second-line detection rates is shown graphically in Fig 2 below.

Table 4 shows the results of the break-even analysis. Firstly, we find that across all scenarios, that the use of NGS as first-line investigation is effectively a dominant strategy i.e. for any given probabilities of NGS as a first-line investigation, the total expected cost of using NGS as a second-line is higher than using it at first-line over the set of plausible second-line detection rates.

**Table 4:** Results of break-even analysis.

Scenarios	Break-even points (Detection rate of NGS)	
	NGS adjuncts first-line	NGS adjuncts second-line
Baseline	60%	98%
Low infectious disease prevalence	48%	68%
Cost* reduced by 50%	81%	Does not break even
Cost* increased by 100%	39%	50%
Variations of first-line and second-line investigations	51%–78%	82%—does not break even

*Cost of diagnostic tests in infectious disease, solid tumor and unresolved diagnosis

Secondly, even in the baseline case, with a currently reasonable assumption about the cost of testing, the probability of detection at first-line would need to be 60% or greater to be cost-saving relative to current practice. In an environment where the prevalence of infectious disease is low, the likelihood of unresolved diagnosis and overall diagnostics cost are high at baseline. Introducing NGS is hence feasible from a cost perspective at lower levels of diagnostic performance. However, in a low-cost scenario where the diagnostic costs for infectious disease, solid tumour and unresolved diagnosis are reduced, the diagnostic performance of the metagenomic pathogen test needed to increase significantly.

## Discussion

Traditionally, the performance of laboratory investigation is evaluated against analytical criteria based on its clinical use and requirements. Such evaluations include analytical accuracy, precision, sensitivity and specificity. Healthcare resources are limited and care must be taken when introducing an expensive investigation. In this austere environment, better healthcare resource allocation decision can be made when the value of a laboratory test is explicitly weighed against the diagnostic performance and economic impact on the overall diagnostic cost for a particular condition.

This study has some important limitations: of note, we only examined the impact of introducing the metagenomic test to the overall diagnostic cost and excluded costs associated with hospitalization. The high cost of metagenomic test may be justified if it provides faster actionable results thereby reducing the length of hospitalisation, which can return significant cost savings. For some patients, a more definitive answer and shorter turnaround time provided by the metagenomic test compared to sequential infectious disease testing may be a premium worth paying for. A second issue is related to the fact that the decision tree is based on idealised published international guidelines only and in reality, practice differs from these guidelines. However, we note that while these (or any) national guidelines are not universal, they are likely to largely overlap with other clinical guidelines and sufficiently illustrate the application of our methodology. Other assumptions about the performance of the routine diagnostic tests, limiting the cost of metagenomic test to SGD 100–1000, and the lack of further work-up after positive pathogen detection by the metagenomic test could be challenged. However, these assumptions were necessary to keep the model within reasonable limits. In reality, the investigation of PUO is likely to be driven by the clinical presentation and findings of the initial investigations. For patients with clinically apparent diagnosis, a conventional targeted investigation (e.g. polymerase chain reaction, PCR-based test) will yield higher diagnostic return at lower cost. Furthermore, the diagnostic yield of the metagenomic test is likely to be dependent on the type of infection, the sample type, the analytical workflow and the bioinformatics. It may be technically challenging to have an optimised analytical workflow that can detect all pathogens with equally high rates. Finally, metagenomic testing may be a necessary last resort in patients who has exhausted all diagnostic tests or the investigation of a novel outbreak. In spite of these limitations, we believe this simplified presentation lends itself to the illustration of the key insights of our paper.

At present, the reagent cost of next-generation sequencing for metagenomic testing is very high (~SGD 2800 for a run of 5 samples), owing to the need to maximize the analytical sensitivity of the test, which will require extensive sample preparation and high sequencing depth. The actual price the patients can expect to pay will be much higher when other laboratory consumables, overheads, depreciation costs, administrative charges and margins are included. Moreover, it is expected that many patients will need to be tested for multiple sample types to maximize the odds of detecting the pathogens. It is also likely that an analytical run may be performed before other patient samples are received to minimize the turnaround time for patients with PUO. Based on the analysis in this study, it is unlikely that the metagenomic test can achieve the break-even diagnostic costs in the setting of PUO currently.

A PUO by definition must have been present for at least three weeks of symptoms and three days of investigations [1]. During this time, the patient would have undergone most of the routine first- and second-line investigations. In practice, the metagenomic test will most likely be used as an adjunct investigation, after failing the first- and second-line investigations, when the opportunity for cost savings is greatly diminished. As a result, the detection rate needs to be very high (51%-78%) in order to remain cost-neutral. This is approximately 2.5–4 times the diagnostic yield reported for CT scan of the abdomen/ pelvis (20%) for the investigation of PUO [23].

The ability for metagenomic test to achieve such a high detection rate is questionable as routine blood and urine cultures have been shown to have extremely low yields [22]. Nevertheless, the advantage of the metagenomic test is that it does not rely on living organism to yield a positive result. In patients who have received prior antimicrobial therapy, they are much more likely to return negative culture results. However, the genetic material of the pathogen may still be detectable by metagenomic testing in such patients. On the other hand, when the detection rate requirement is set to minimum, the cost of the metagenomic test needs to be very (unrealistically) low in order to remain cost-neutral to the healthcare system.

For a molecular test like a metagenomic test that is expensive but claims to be ‘catch all’, its clinical benefit lies in its early application in the diagnostic pathway, when molecular targets are at their highest concentrations, i.e. during the acute phase of the illness. One possible way to overcome this challenge is to archive sufficient and appropriate clinical samples early on when the patient presents to the healthcare system. This practice is not routine at present, and will need to be built into the overall clinical workflow. In addition, the metagenomic test needs to be applied to the correct sample type for the pathogen in question, but this cannot be selected if the pathogen is unknown. So potentially, several different sample types may need to be tested using the metagenomic test, further increasing the cost, before a relevant ‘hit’ is registered—with ‘relevant’ meaning a pathogen that can give rise to the range of clinical symptoms matching those with which the patient presents.

By the time of a diagnosis of PUO is made (third week of fever) [1], most circulating pathogen is likely to have disappeared, and serological ‘convalescent’ testing would now be the main option. There are exceptions to this though, for certain pathogens, if the right sample can be tested, e.g. norovirus, enterovirus, rotavirus may all be shed in stool for several weeks after recovery from the acute infection [27–29]. Adenovirus may continue to be shed in upper respiratory or gastrointestinal tract samples for many weeks after acute or reactivated infection in children or the immunocompromised [30–32]. After acute cytomegalovirus infection, the virus can be shed for weeks in the urine and blood of preterm infants who usually acquire post-natally via breast-feeding [33]. For Zika virus, we now know that the virus can continue to be shed in the semen for over 100 days after the acute infection [34, 35]. Serological testing is thus complementary to such acute molecular tests, as antibodies can typically take 1–2 weeks to appear, by which time the acute viremia may have passed, leading to low detection yield by molecular assay.

The use of the metagenomic test in anything other than a first-line screening risks a very low yield and poor sensitivity. This then poses the question: can the metagenomic test actually be used in clinically ambiguous patients to then prevent them progressing to PUO status, by making a definitive diagnosis earlier? To some extent, this depends very much on either the admitting clinical team having absolutely no idea about any differential diagnoses that they can test conventionally, or the clinical team being ignorant or passive in the extreme and not even bothering to consider any possible differential diagnoses to test—both of which are highly unlikely.

The other scenario is for the admitting team to be able to exclude all the likely diagnoses quickly enough to allow the metagenomic test to be used early enough, whilst sufficient molecular targets are circulating in the patient, to make a reliable diagnosis, e.g. within five days. This is also highly unlikely. Beyond 1–2 weeks when the definition of the PUO then starts to apply to the illness, apart from the prolonged viral shedding exceptions above, serology then becomes the more effective diagnostic.

Another facet of this metagenomic test analysis is whether an early negative result on the metagenomic test would then prompt the clinical team to stop looking for an infectious disease diagnosis and consider an oncological one instead. This is quite a different application of the assay, changing it from a rule-in assay for helping to diagnose an infectious disease cause, to a rule-out assay to then permit other non-infectious causes to be considered. The former relies on a high positive predictive value (i.e. TP/(TP+FP) = 1.0), whereas the latter on a high negative predictive value (i.e. TN/(TN+FN) = 1.0). To perform well on both counts is extremely demanding (and therefore unlikely) for any assay where the background prevalence of the disease may be either variable or unknown.

Pyrexia of unknown origin remains a largely under-studied condition. The precise prevalence of PUO remains uncertain due to the constant improvement in imaging and biomarker investigations [36]. Data on healthcare costs, burden of illness costs, patient level functioning and activities of daily living of affected individuals are important to allow assessment of the impact any diagnostic or therapeutic intervention may have on this condition and clearly deserves more attention.

Overall, the analysis in this study can only be applied to infectious agents that may still be shedding after more than two weeks, after a diagnosis of PUO is instigated, which can then still be detected with a reasonable sensitivity on a metagenomic test. This analytical process, however, has yielded perhaps far more useful insight as to how and where such an assay should be used—perhaps not as a fall-back assay for investigating PUO cases, but more of a frontline acute diagnostic test when there are high concentrations of the molecular target circulating in the patient, if the appropriate sample types are selected for metagenomic testing.
